# Novel Fat Replacers Based on Pork Lard and a Cold Gelling System in the Reformulation of Reduced-Fat Fresh Pork Sausages Containing Silicon from Diatomaceous Earth Powder

**DOI:** 10.3390/gels11080618

**Published:** 2025-08-08

**Authors:** María Dolores Álvarez, Arancha Saiz, Susana Cofrades

**Affiliations:** Institute of Food Science, Technology and Nutrition (ICTAN-CSIC), 28040 Madrid, Spain; a.saiz@ictan.csic.es

**Keywords:** pork lard, emulsion gel, fat bulking agent, fresh pork sausages, silicon, in vitro gastrointestinal digestion

## Abstract

This study examines the effects of an emulsion gel (EG) and a fat bulking agent (BA), both formulated with pork lard (PL) and an alginate-based gelling system, as animal fat replacers in the reformulation of reduced-fat fresh pork sausages. Both fat analogs were characterized in terms of texture, color, and in vitro gastrointestinal digestion (GID) before being used in the reformulation of four fresh pork sausages, without and with added silicon (Si) from diatomaceous earth powder: S/EG, S/EG-Si, S/BA, and S/BA-Si. Reduced-fat sausages elaborated exclusively with pork backfat (PB), without and with Si, were used as controls (S/C and S/C-Si). Both EG and BA showed adequate physicochemical characteristics and slowed in vitro GID compared to PL and PB. Replacing 75% PB with EG or BA did not negatively impact the technological, nutritional, or sensory properties of the reformulated pork sausages, which were kept for 14 days under refrigeration. Additionally, sausages containing EG or BA as fat substitutes presented lower lipid digestibility after in vitro digestion compared to the control samples. The addition of Si further limited fat digestion, as reflected by the lower release of free fatty acids after in vitro GID compared to products without added Si. This effect was more pronounced in EG-based formulations. Therefore, the use of EG as a PB replacer, together with the addition of Si, could become a promising strategy for developing healthier meat products. This finding may have important implications for the development of functional meat products aimed at reducing fat content and lipid absorption, thereby laying the foundation for precision nutrition strategies focused on improving individual health outcomes.

## 1. Introduction

Fresh pork sausages are a highly popular processed meat product, widely consumed for their affordability, ease of preparation, and notable nutritional benefits. With approximately 60% meat content, they provide proteins of biological value, essential B vitamins, and highly bioavailable minerals, among others. However, these products raise health concerns due to their elevated animal fat content (over 27%), particularly saturated fats. Excessive consumption of cholesterol and saturated fats has been associated with negative health effects (e.g., cardiovascular disease). Nevertheless, saturated fats play a crucial role in the technological properties and sensory characteristics of this type of products. To address these concerns, the meat industry has been searching for fat analogs that not only improve the nutritional profile of fat content but also simulate the characteristics of saturated fats. Recent research has focused on structuring healthy oils of vegetable or marine origin to create oleogels (OGs), emulsions gels (EGs), and oil bulking agents (BAs) [1] to be used as animal fat replacers. Generally, EGs are defined as complex colloidal materials where emulsions and gels (hydrogels) coexist. By contrast, BAs are based on the dispersion of a large amount of oil droplets in a continuous aqueous matrix-forming gel. In this regard, alginate gels present interesting opportunities as fat bulking agents for their ability to form gels in the presence of calcium salt [2]. Alginate gels involve polymeric molecules that are cross-linked to form a rigid three-dimensional macromolecular network containing a great proportion of water within its structure, presenting solid structures with characteristics close to those of the animal fat they are intended to replace. While EGs consist mostly of proteins (emulsifying agent) and hydrocolloids (gelling agent), BAs comprise polysaccharides as gelling agents. The incorporation of these lipid materials has improved the lipid content of numerous reformulated meat products, but it has also led to processing issues, such as fatting out and oil exudation. These defects often compromise the desired quality parameters of the reformulated products [3,4]. To overcome these issues, there is a new strategy based on modifying animal fat itself by developing emulsions with pork lard (PL), which has a balanced lipid profile between saturated and unsaturated fats. Furthermore, Siri-Tarino et al. [5] concluded that there is insufficient evidence to definitively link saturated fat intake with cardiovascular disease. In this context, the development of gelled PL lipid materials, such as EGs and fat-based BAs, has emerged as an ideal approach to replace animal fats. These gelled structures not only have a lower fat content than PL but also exhibit significant cross-linking, which hinders hydrolytic enzymes from accessing fat droplets, therefore reducing lipid digestibility [6,7]. While EGs and OGs have been previously used as fat analogs to reduce lipid content and absorption, BAs have only been used to improve the lipid content of meat products, rather than to limit fat digestibility.

Another approach to reformulating healthier meat derivatives involves the incorporation of bioactive compounds. Silicon (Si) is an essential micronutrient known for its important health effects, including its role as a bone mineralization inducer and neuroprotector. Also, and as evidenced in animal experimental studies, Si exhibits other lesser-known characteristics, including antioxidant activity, as well as hypoglycemic and hypolipemic properties [8]. In this context, Si has been incorporated into emulsions stabilized with a protein and cellulose ether mixture, showing an important reduction or delay in lipid digestibility at the end of GID compared to emulsions without Si [9]. The Si-containing emulsions were then used to completely replace pork backfat (PB) in the reformulation of reduced-fat pâtés, resulting in lower fatty acid (FA) contents in the bioaccessible phase compared to their counterparts without Si [10].

To date, there are no studies comparing two types of fat analogs elaborated with PL (EG vs. BA) for their subsequent incorporation into widely consumed processed meat products. Therefore, the objective of this study was to formulate two different lipid materials using PL and the alginate-based gelling system, to be employed as PB substitutes in reduced-fat fresh pork sausages, without and with added Si as a bioactive compound. For this purpose, the study evaluated the technological, nutritional, microbial, sensory characteristics, as well as lipid digestibility after in vitro GID of the fresh pork sausages.

## 2. Results and Discussion

### 2.1. Visual Appearance, Physicochemical Characteristics, and Lipolysis During In Vitro GID of Pork Backfat, Pork Lard, and Fat Analogs

**Figure 1:**
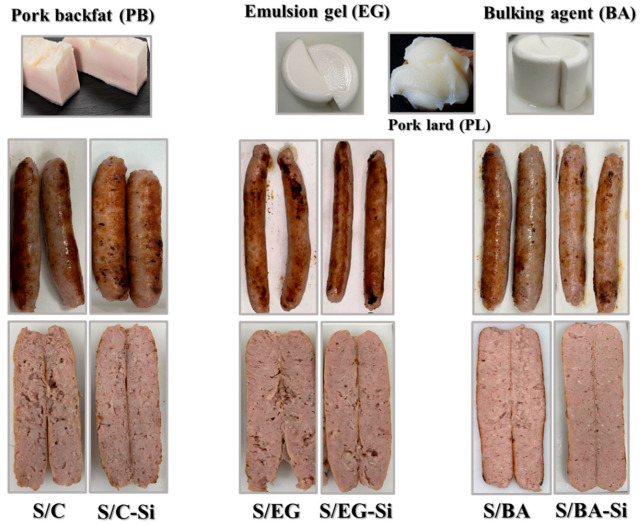
Visual appearance of lipid materials: pork backfat (PB), pork lard (PL), the emulsion gel (EG), and the fat bulking agent (BA) as fat analogs and their application in different pork sausages. EG, gel emulsion containing sodium caseinate as the emulsifying protein and the cold gelling system based on sodium alginate (SA); BA, fat bulking agent containing SA as the cold gelling system; S/C and S/C-Si: control sausages elaborated only with PB without and with added Si, respectively. S/EG and S/EG-Si, sausages with 75% substitution of PB with EG without and with added Si, respectively; S/BA and S/BA-Si, sausages with 75% substitution of PB with BA without and with added Si, respectively.

Figure 1 shows the visual appearance of pork backfat (PB), pork lard (PL), and the two fat analogs: the emulsion gel (EG) and the fat bulking agent (BA). Both fat analogs exhibited a similar whitish appearance at room temperature, visually resembling PL but slightly different from PB. In addition, the incorporation of the gelling system—formed by sodium alginate, CaSO_4_, and pyrophosphate—promoted the gelation process in EG and BA, conferring a homogeneous appearance and solid-like characteristics more similar to those of PB than those of PL (Figure 1).

In terms of texture, both fat analogs exhibited a maximum breaking force (Figure S1) typical of gel structures. However, the textural properties of the EG sample were significantly lower than those of BA (Table 1). By contrast, neither PB nor PL presented a maximum force peak, as their structures did not break during the test, and PB had much higher penetration parameter values at the end of the test compared to PL (Figure S1).

Due to these differences, the textural parameters of PB and PL were not statistically compared to those of EG and BA (Table 1). The textural differences observed between EG and BA may be attributed to variations in oil droplet size resulting from their respective preparation processes. In this sense, the creation of a bulking system, such as BA, implies the dispersion and physical entrapment of oil droplets within a hydrogel matrix, forming a structured system without emulsifiers. As a result, BA features larger oil droplet sizes compared to conventional gelled emulsions, where the emulsification process with an emulsifying protein, in our case SC, leads to smaller droplets. These texture results are consistent with those reported for oil-in-water (O/W) emulsions formulated with proteins [11] and for O/W emulsions stabilized with SC, without and with microbial transglutaminase (MTG) [12]. Other authors have examined the stability of O/W emulsions containing caseinate-coated droplets by adding sodium alginate [13]. These authors observed that, at pH 6 and 7, alginate did not adsorb to the droplet surfaces due to electrostatic repulsion between anionic groups, either on the alginate or on the adsorbed caseinate (far from the isoelectric point of the protein). Similar electrostatic repulsions between negatively charged droplets and alginate molecules could explain the lower textural gel properties of EG compared to BA (Table 1).

Regarding the objective color parameters (Table 1), the L* and b* values were significantly higher in EG and BA than in PB and PL, with EG displaying the highest L* and b* values. By contrast, PB exhibited the highest a* value, lower brightness, and a greater tendency toward red. This is likely due to the presence of traces of meat and other biological components in this raw material (Figure 1; Table 1), while the higher L* and b* values of EG compared to BA could be attributed to the presence of SC as the emulsifying protein.

**Figure 2:**
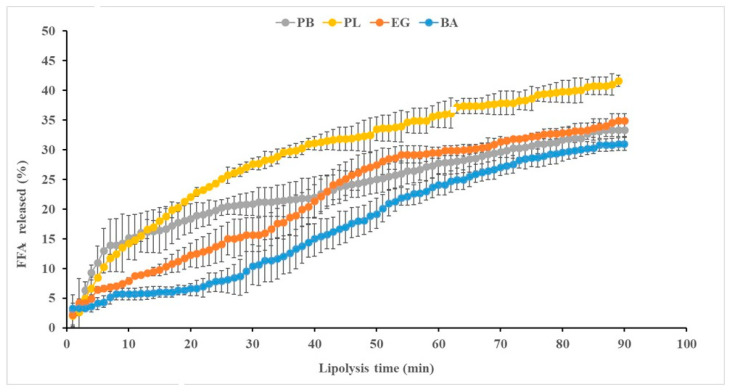
Profiles of the free fatty acids (FFA) released from the different lipid materials (PB, PL, EG, and BA) with lipolysis time. For sample denomination, see Figure 1.

Figure 2 illustrates the rate of FFA release corresponding to PB, PL, EG, and BA during lipolysis. Both PB and PL exhibited a similar trend in FFA release during the first 13 min of intestinal digestion. However, after this period, the FFA release from PB was slower than that from PL, reaching lipolysis levels of ~33% and ~41.61%, respectively, after 90 min. These differences could be attributed to variations in the physical properties in PB and PL fatty materials as a result of the clarification process to obtain PL from PB. During clarification, changes in the positional distribution of triacylglycerols (TAGs) result in PL having a semi-solid consistency, a lower melting point, and a lower solid fat content compared to PB at the GID temperature. Fats with lower melting points have higher in vitro digestibility and are more easily absorbed over prolonged digestion. In this context, the highly digestible PL exhibited a less compact microstructure and smaller fat crystals than PB. Thus, PB was digested less efficiently, as fats containing stearoyl-richer TAGs require higher temperatures to melt [14].

Regarding the lipolysis of the fat analogs (Figure 2), both showed a lower release of FFA during the first 40 min of digestion compared to PB and PL, with BA exhibiting a lower FFA release than EG. This was followed by a gradual and similar increase in the release profile over time, although some differences between the two persisted. While EG showed a rapid increase in FFA release, BA exhibited a significant delay in lipolysis until min 70, whereas up to min 90 of digestion, the differences in FFA release decreased. Notably, at the end of intestinal digestion, the percentages of FFA released from both fat analogs and PB were very similar (ranging from 31–35%) and significantly lower than those from PL. These lipolysis percentages were lower than those previously reported for emulsions stabilized with soy protein concentrate but without gelation [9], as well as for other gelled emulsions formulated with MTG and ĸ-carrageenan [6]. On the other hand, the initial delay in lipolysis observed in BA could be attributed to its higher consistency (Table 1), as softer gels like EG would degrade faster during in vitro GID than harder gels like BA [15]. These authors reported that harder gels have a denser, more compact particulate gel structure with increased cross-linking, making it more difficult for hydrolytic enzymes to access fat droplets. In addition, the higher rate and extent of lipolysis observed in EG could be associated with the fact that emulsifying proteins such as casein, which are commonly used in food-grade emulsions, form an interfacial protein film that can be easily displaced by bile salts, thereby facilitating lipid digestion by lipases [16].

### 2.2. Characterization of Reformulated Fresh/Cooked Sausages

To justify the use of animal-based analogs in this study, it should be noted that animal fat plays a key role in maintaining product quality and sensory acceptance. It also presents a well-balanced lipid profile between saturated (SFA) and unsaturated fatty acids (MUFA and PUFA), with oleic acid being the predominant fatty acid. Moreover, its lipid composition makes it less susceptible to lipid oxidation compared to vegetable and marine oils commonly used in previously studied animal fat replacers. In this context, the fatty acids that most effectively improve the lipid profile of meat products are alpha-linolenic acid (ALA), docosahexaenoic acid (DHA), and eicosapentaenoic acid (EPA), which are primarily found in seeds and fish. However, the quality characteristics—particularly the sensory attributes—of reformulated products with oils are often negatively affected when compared to those made with animal fat in emulsion-type analogs. Additionally, the high polyunsaturated fatty acid content of vegetable and marine oils makes them highly prone to lipid oxidation. In this context, a promising strategy to develop healthier meat products without compromising sensory quality could involve reducing lipid digestibility in whole animal fat-based formulations.

#### 2.2.1. Proximate Composition and Energy Content

Table 2 presents the proximate composition of the cooked sausages formulated with PB alone (S/C and S/C-Si), with the emulsion gel (S/EG and S/EG-Si), and with the fat bulking agent (S/BA and S/BA-Si), both of which were used as partial PB replacers at a high substitution percentage (75%). All fresh sausages were formulated with a theoretical reduced-fat content (~13.43%) and a protein content ranging from 12.48 to 13.94%. The partial substitution of PB with EG or BA, along with the incorporation of Si, resulted in significant modifications in the proximate composition of the cooked sausages (Table 2). The sausages formulated without Si (S/C, S/EG, and S/BA) exhibited higher moisture content than their counterparts containing Si (S/C-Si, S/EG-Si, and S/BA-Si), which was expected given that Si was added by replacing water. Also, and as anticipated, the cooking process induced an increase in protein content across all samples, particularly in both control sausages (Table 2). Conversely, the fat content of the control sausages was significantly lower than that of sausages containing EG and BA as fat replacers, with no significant differences observed between the latter. In addition, samples containing Si exhibited a significantly higher ash content.

Given that fresh commercial pork sausages typically contain ~30% fat, all of the reformulated samples qualified as reduced-fat sausages (fat content ranging from 13.11 to 15.87%). Consequently, their energy content (from 192.79 to 209.47 kcal/100 g product) (Table 2) was lower than that of commercial and other formulated pork sausages (~325 kcal/100 g product) [17,18].

#### 2.2.2. Physicochemical Properties

**Figure 3:**
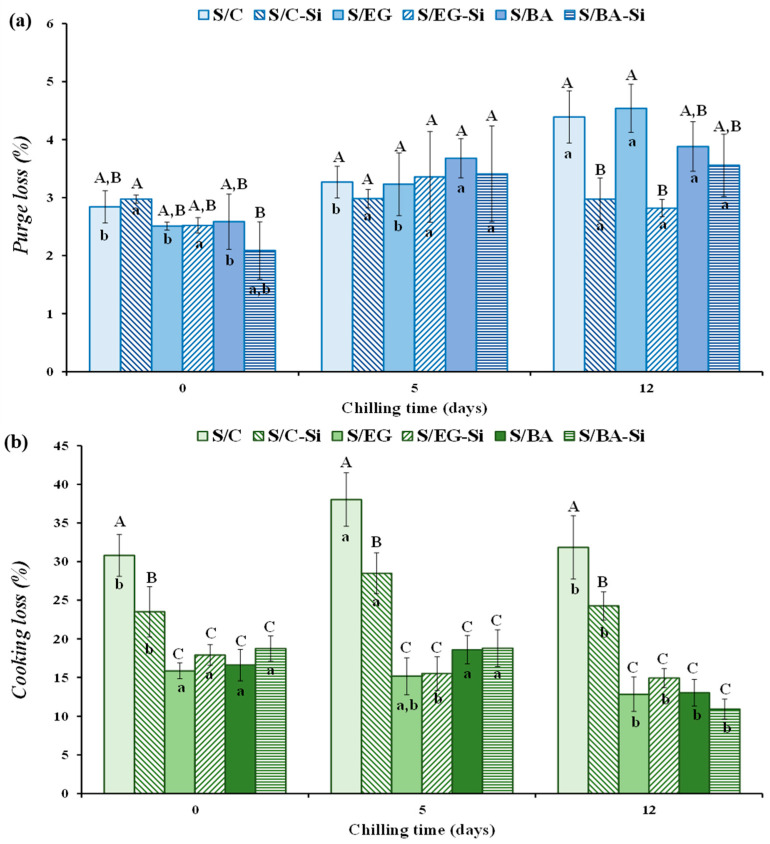
Binding properties as measured by the (**a**) purge loss and (**b**) cooking loss of the reduced-fat pork sausages during chilled storage. For sample denomination see Figure 1. A–C Different letters indicate significant differences among sample groups. a,b Different letters indicate significant differences among storage days.

The processing losses in the fresh sausages were minimal, ranging from 1.45 to 2.52% with no significant differences among formulations. The purge and cooking losses during chilled storage are shown in Figure 3a,b, respectively. The purge loss varied between 2.09% and 4.54% and, in general, up to 5 days of storage, neither the partial substitution of PB with EG or BA nor the incorporation of Si had a significant effect on this parameter. All samples exhibited similar exudate values (Figure 3a). However, after 12 days of chilled storage, the purge loss increased in all samples without added Si (S/C, S/EG, and S/BA) compared to values recorded at days 0 and 5. By contrast, the purge loss in sausages with added Si (S/C-Si, S/EG-Si, and S/BA-Si) remained stable throughout the storage period, indicating that Si reduced exudate losses at the end of the storage period.

Regarding cooking loss, it is well known that the cooking process of meat products results in significant mass loss, primarily influenced (*p* < 0.05) by formulation and, to a lesser extent, by storage time (Figure 3b). Although reported weight losses during sausage cooking vary considerably in the literature, the values recorded in this experiment (ranging from 11% to 38%) fall within the normal range (15% to 40%) for comparable ground meat products [19]. The highest cooking losses were observed in the control samples (S/C and S/C-Si) throughout the entire storage period (0, 5, and 12 days) (Figure 3b). In this case, the presence of Si reduced cooking losses only in the control sample (S/C-Si vs. S/C). The partial substitution of PB with either EG or BA resulted in a significant reduction in cooking loss, with no differences between the values obtained for sausages containing these two fat analogs. This suggests that relatively high proportions of the fat and water present in both fat analogs were effectively retained after thermal treatment. By contrast, the presence of Si resulted in a loss-reducing effect only in formulations in which losses were substantial (control samples). These differences could be attributed to the fact that, although all cooked sausages had very similar moisture, the protein and fat contents (Table 2) of formulations containing EG and BA retained water more effectively within their gel structure, whereas the free water added during the formulation of S/C and S/C-Si was less well retained. The processing, purge, and cooking losses observed in this study are consistent with those reported by other authors for pork sausages [17,20]. The latter authors found that replacing PB with chia and oat emulsion gels in reduced-fat sausages significantly reduced cooking loss.

**Figure 4:**
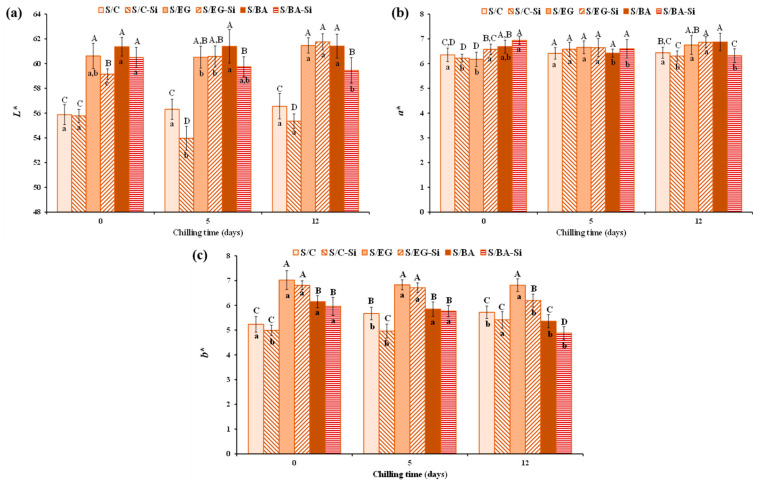
Color parameters (**a**) L*, (**b**) a*, and (**c**) b* of the reduced-fat pork sausages during chilled storage. For sample denomination see Figure 1. A–D Different letters indicate significant differences among sample groups. a–c Different letters indicate significant differences among storage days.

The color parameters of the cooked sausages are shown in Figure 4. The partial replacement of PB with EG or BA increased lightness (L*) and yellowness (b*) values, which remained relatively stable throughout the storage period (Figure 4a,c).

The cooked sausages containing BA (S/BA) exhibited significantly (*p* < 0.05) lower b* values compared to those containing EG (S/EG) (Figure 4c). This is consistent with the b* values observed in both BA and EG (Table 1). The effect of adding Si on the color parameters depended on the type of fat analog used but, in general, Si tended to reduce L* values. However, only minor differences were found in a* values as a consequence of either formulation or chilled storage, with values ranging from 6.17 to 6.94. The differences in L* and b* values can be attributed to variations in color and composition of PB and PL compared to EG and BA. Specifically, PB contained approx. 86% fat and may have also contained trace amounts of meat and very little water content (~5%), whereas the fat analogs contained clarified PL, which is devoid of meat residues and has a high water content (60%). Despite the significant differences found in L* and b* parameters in the cooked sausages, their technological relevance is negligible, as evidenced by the visual appearance (Figure 1) and the sensory evaluation described below. Similar changes in pork sausages resulting from PB replacement with different fat analog (emulsions, oil bulking agents, etc.) have been previously reported [17,20].

**Figure 5:**
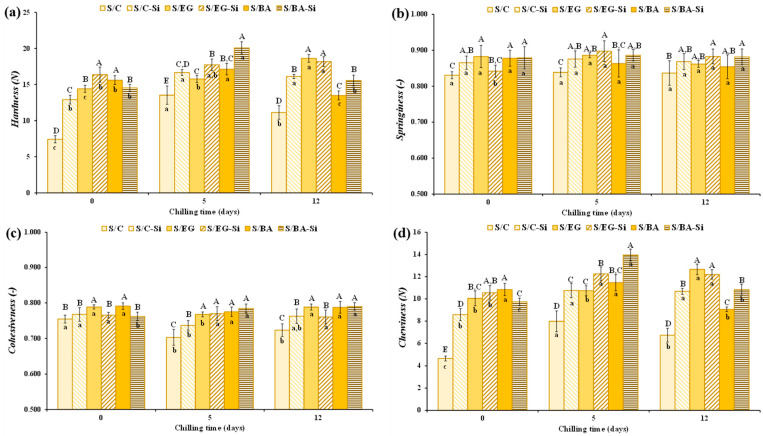
Textural parameters (**a**) hardness, (**b**) cohesiveness, (**c**) springiness, and (**d**) chewiness of the reduced-fat pork sausages during chilled storage. For emulsion denomination see Figure 1. A–D Different letters indicate significant differences among sample groups. a–c Different letters indicate significant differences among storage days.

The textural properties of the cooked sausages were significantly (*p* < 0.05) affected by formulation and storage time (Figure 5). However, variations in springiness and cohesiveness were minimal, with values ranging from 0.831 to 0.898 and from 0.703 to 0.790, respectively (Figure 5b,c). Hardness and chewiness, which were positive and significantly correlated, were significantly lower in the control sausages formulated with PB compared to those prepared with EG or BA throughout the storage period (Figure 5a,d). The increased hardness observed in the sausages containing EG or BA can be attributed to the fact that the components included in these fat analogs (mainly water and fat) were better retained within the meat protein gel during cooking compared to the control sausages with PB. This contributed to the higher hardness and chewiness of these products [21]. The reduction in cooking loss caused by Si in the control samples (S/C-Si vs. S/C; Figure 3b) caused a significant increase in the textural properties at 0, 5, and 12 days of chilled storage. In general terms, the presence of Si tended to increase the hardness of the sausages compared to their counterparts without added Si. Furthermore, hardness and chewiness also increased with increasing storage time, although the magnitude of change (typically minor) and the specific time points at which it occurred (5 or 12 days) varied depending on the fat analog used, probably due to a slight water loss (Figure 5a,d). A similar textural behavior has been observed in different meat products, including cooked pork sausages, in which different fat analogs, such as gelled emulsions or oil bulking agents, were used as animal fat replacers [22,23].

#### 2.2.3. Lipid Oxidation

**Figure 6:**
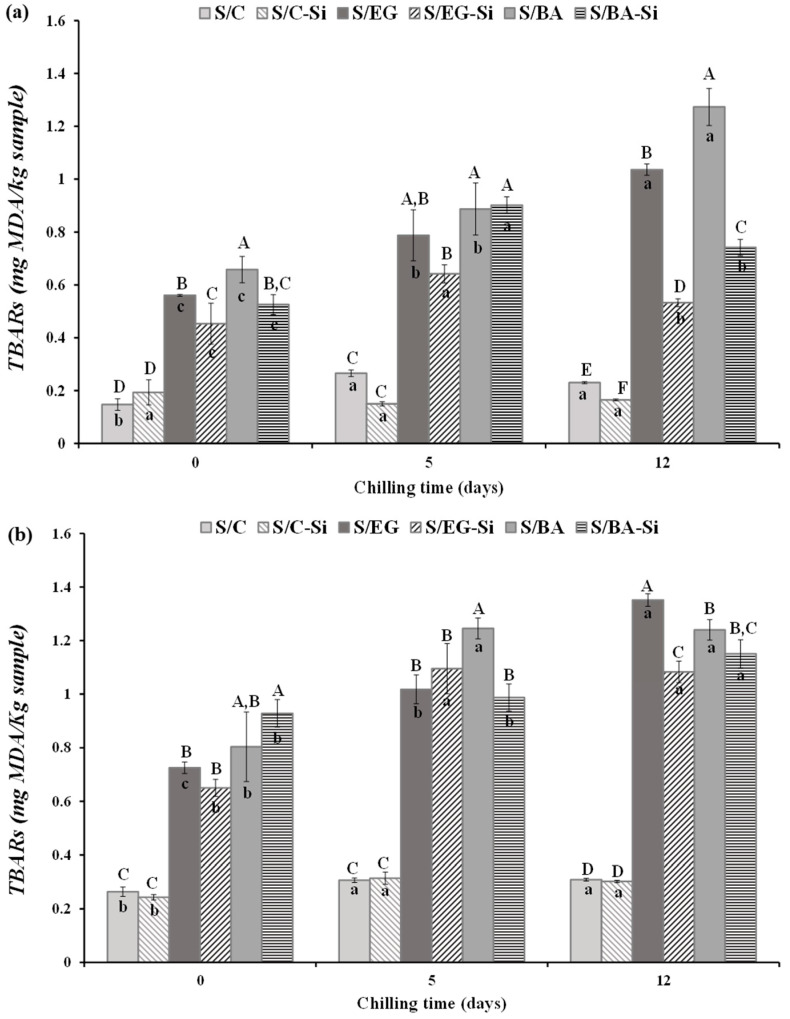
TBARS values of the (**a**) fresh and (**b**) cooked reduced-fat pork sausages during chilled storage. For emulsion denomination see Figure 1. A–F Different letters indicate significant differences among sample groups. a–c Different letters indicate significant differences among storage days.

The results of lipid oxidation, assessed by malondialdehyde levels (TBARS), in fresh and cooked sausages during chilled storage are shown in Figure 6. Lipid oxidation was significantly lower in fresh sausages (Figure 6a) compared to cooked sausages (Figure 6b), which aligns with the well-known fact that thermal processing increases lipid oxidation [24]. The replacement of PB with either EG or BA caused significant increases in TBARS values in both fresh and cooked samples (*p* < 0.05), with no significant differences between EG and BA. These results were expected, as the control sausages contained only PB, which did not undergo any treatment, thus exhibiting low susceptibility to oxidation. Conversely, the sausages formulated with EG or BA contained PL, obtained from the above-mentioned PB clarification process (heating at ~100 °C for at least 30 min). This heat treatment likely explains the higher TBARS values found in the sausages containing fat analogs, since the PB clarification process used to obtain PL (100 °C for at least 30 min) does not alter the lipid profile. Initially, the addition of Si had no effect on the oxidation levels in the cooked samples, while in the fresh samples, Si was associated with reduced TBARS levels in the reformulated sausages compared to their counterparts without Si.

The changes in TBARS levels during storage were formulation dependent, and although they were significant, they were not particularly relevant in the case of S/C and S/C-Si. However, different behavior in terms of lipid oxidation during storage was observed in the reformulated products containing EG or BA. In these products, the highest TBARS values were detected after 12 days of storage, particularly in S/EG. Notably, in this sample, Si had a significant antioxidative effect, reducing oxidation levels (Figure 6b). These results are consistent with those observed by other authors in meat products formulated with pork fat and remain below the reported threshold for sensory detection of undesirable flavors in processed meat products [25,26].

#### 2.2.4. Sensory Analysis

Overall, the sensory evaluation of pork sausages was unaffected by formulation (Table 3). Panelists were unable to distinguish (*p* > 0.05) among samples in terms of appearance, color, odor, or texture acceptability. The only significant difference was observed in flavor acceptability and overall acceptability, where S/BA received lower scores than S/C-Si. However, there were very little differences in the overall acceptability of the different sausages, regardless the presence of Si or the substitution of PB with a fat analog (Table 3), either in the form of a gelled emulsion or a bulking agent (except for S/BA, which was rated lower in acceptability compared to S/C-Si). These results are consistent with those obtained in a pâté formulated with biopolymeric emulsions containing PL, which exhibited sensory attributes comparable to the control pâtés [10]. Similar results have also been reported in burger patties reformulated with an EG containing polyunsaturated oil as a PB replacer, where no significant differences in sensory attributes were observed [27].

#### 2.2.5. Microbiological Analysis

The microbiological counts of the fresh sausages are shown in Table 4. Initially, the control samples (S/C and S/C-Si) exhibited the lowest levels of total viable count (TVC), lactic acid bacteria (LAB), and *Enterobacteriaceae,* with values ranging from 5.95–6.31, 3.95–3.86, and 2.75–2.63 Log cfu/g, respectively. These values align with those reported by other authors for similar products [17,28,29] and, as counts were below 6 Log cfu/g, are acceptable according to the total microbial quality standard for pork sausages and ground beef [30]. This is noteworthy given the fact that sausage preparation involves a high level of handling of all raw materials, increasing susceptibility to contamination [28]. However, the partial replacement of PB with BA, and particularly with EG in the reformulated sausages (S/EG, S/EG-Si, S/BA, and S/BA-Si), caused significant increases in TVC, LAB, and *Enterobacteriaceae* levels. These increases could be attributed to the manual processing of PL during clarification, in contrast to the control products, which were prepared using PB without additional handling.

During chilled storage (Table 4) and under vacuum packaging, the microbiological counts remained relatively stable across all groups of refrigerated fresh sausages. In this regard, slight yet significant increases in TVC and LAB levels in the control sausages were observed after 5 days of storage; however, the values remained constant until the end of the storage period. These increases are associated with protein and fat degradation occurring during storage mainly due to endogenous meat enzymes. However, the observed microbial growth was generally lower than that observed in similar fresh products [29,30], where after 3–5 days of storage, levels of 8 Log cfu/g were reported. Nevertheless, the TVC, LBA, and *Enterobacteriaceae* levels in the reformulated sausages with EG or BA, without and with added Si, hardly changed throughout the storage period (Table 4). The low microbial growth rate observed across all samples during storage may be mainly attributed to the preservative effects of vacuum packaging in combination with low storage temperatures.

### 2.3. Total FFA Released from Digested Cooked Sausages

The total FFA (mg/g fat) released after in vitro GID ranged from 454.8 to 554.0 mg FFA/g fat, with the highest values observed in the S/C sample and the lowest values observed in the S/EG-Si product (Table 5).

**Figure 7:**
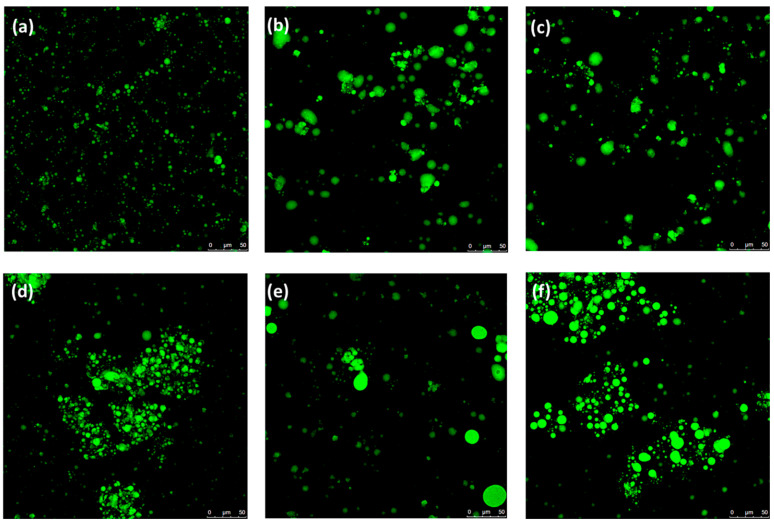
Confocal micrographs of the digests of the six different cooked sausages after in vitro gastrointestinal digestion (GID). Scale bar = 50 µm. (**a**,**d**) Control sausages (S/C and S/C-Si) without and with added silicon (Si), respectively; (**b**,**e**) sausages with 75% substitution of PB with EG (S/EG and S/EG-Si) without and with added Si, respectively; (**c**,**f**) sausages with 75% substitution of PB with BA (S/BA and S/BA-Si) without and with added Si, respectively.

These findings are consistent with those reported by Asensio-Grau et al. [31] for different meat matrices (hamburger, sausage, etc.) after in vitro GID under similar duodenal conditions. The replacement of PB added as bulk fat in the control samples with either of the fat analogs (EG or BA) resulted in a similar reduction in FFA release. As all samples are classified as coarsely processed meat products, the differences found between them can be attributed to variations in the structural organization of the lipid material added during processing, leading to distinct fat distribution patterns in the final products. Previous studies have suggested that lipid bioavailability depends on physicochemical properties as well as on the structural organization of lipids in the matrix [32]. In this context, the lower amount of FFA released after in vitro GID in sausages where PB was replaced with EG or BA is presumably due to the embedding of fat within a gel matrix for both fat analogs. As a result, although partially digested in the gastric and intestinal phase, the oil droplets would remain trapped within the gel network and dispersed in the meat matrix. This structural constraint limits exposure to bile and lipase activity, thus delaying digestion. This result highlights the role of lipid structural organization in limiting the degree of digestion. To confirm this hypothesis, microscopic evaluation of the samples was performed following intestinal digestion (Figure 7). The digests of the samples containing EG or BA (Figure 7b,c) exhibited larger oil droplets, probably due to the reduced lipid digestibility caused by restricted enzymatic access to the surface of the oil droplets embedded in the gel matrices. By contrast, the digest of S/C (Figure 7a) displayed smaller oil droplets, as the droplets from bulk fat are more accessible to lipase. This is consistent with results found by Diao et al. [33]. However, the different structural characteristics of EG and BA (Figure 7) had a minimal impact on lipid digestion (Table 5).

In addition, the presence of Si in the sausages delayed lipid digestion, with the samples containing Si presenting lower FFA release than in their counterparts without Si (Table 5). The most significant effect was observed in S/EG-Si. This could be due to the partial digestion of the emulsifying protein (SC) in EG during the gastric and intestinal phases, leading to the formation of smaller oil droplets compared to those confined in the bulk fat of BA. Consequently, Si may have further delayed lipid digestion. Previous studies have reported the ability of Si to reduce lipolysis in both biopolymeric emulsions and pâtés elaborated with these systems [10]. These results are supported by the microscopic images, where larger oil droplets were observed in S/EG-Si (Figure 7e) and S/BA-Si (Figure 7f) compared to S/C-Si (Figure 7d).

### 2.4. FA Profile of Undigested and Digested Cooked Sausages

Table 5 presents the main FA content of the reduced-fat cooked pork sausages before and after in vitro GID. As all sausages were produced with the same fat type and content, and considering that Si addition does not affect the lipid profile, the reported data for the FA profiles of the cooked, undigested samples represent the mean values of all six sausage types. Oleic acid (monounsaturated) was the most abundant FA in all samples, followed by palmitic and stearic acids (saturated), linoleic acid (polyunsaturated) and vaccenic acid (monounsaturated), in that order, comprising about 90% of total FA in TAG form. These results align with FA profiles found in meat and fat in other pork meat products [34,35].

Regarding the main FA content released after in vitro GID of all cooked sausages (Table 5), oleic acid was released in the highest quantity, followed by stearic, palmitic, linoleic, and vaccenic acids. This shows a strong and positive correlation with the lipid composition of the undigested samples. In general, after 90 min of intestinal digestion, the total release of these five main FA was higher in the control sausage (S/C) than in the samples containing EG or BA, which is consistent with the total FFA results. It should be noted that Si significantly reduced oleic acid solubilization in the micellar phase, with the greatest reduction observed in S/C-Si (13.94% lower than in S/C), followed by S/EG-Si and S/BA-Si (10.84 and 4.29% lower, respectively), as compared to their counterparts S/EG and S/BA. This may once again be attributed to the more homogeneous fat distribution in the control product, whereas fat gelation favors the delaying effect of Si on lipid digestibility.

Additionally, the bioaccessibility (BAC) of the five main FA at the end of in vitro GID of the different pork sausages was calculated (Table 6). BAC values in the pork sausages ranged from 30.0% to 87.6%, with a mean value of 57.8%. These values align with previous digestion studies with fat, where BAC ranges of 50–90% [36] and 34–71% [37] were reported for intestinal FA hydrolysis. Regarding the BAC of the five specific FA studied (Table 6), it is noteworthy that, among the main SFA, stearic acid had greater BAC values than palmitic acid for all six sausage types. Notably, both of these SFA had significantly higher BAC values in control sausages S/C and S/C-Si than in the reformulated samples with EG or BA as PB partial substitutes. The lower BAC value of palmitic acid may offer beneficial health effects by mitigating its potential pro-inflammatory and lipotoxic effects. This could be particularly relevant in individuals with high dietary intake of saturated fats or those prone to metabolic disorders. A lower palmitic acid BAC value may help to reduce the risk of cardiovascular diseases, obesity, and type 2 diabetes. However, stearic acid is considered neutral in this regard. The two main MUFA, vaccenic and oleic acids, displayed very similar BAC values across all samples.

Despite vaccenic acid being a minor FA compared to oleic acid, either before or after in vitro digestion (Table 5), its BAC values were significantly higher in S/C than in the rest of the sausages (Table 6). The effect of Si was particularly notable, since its presence in the formulations significantly reduced the BAC values of most FA, confirming the hypotriglyceridemic effect of Si observed in previous works [6,9].

## 3. Conclusions

Healthy fat-reduced fresh pork sausages were developed using two fat analogs (EG or BA) prepared with pork lard (PL) and a cold gelling system, without and with added Si. The physicochemical, lipid oxidation, and sensory characteristics of the reformulated products closely resembled those of the control samples entirely formulated with pork backfat (PB). In vitro digestion assays, performed on both fat analogs as well as on the pork sausages prepared with EG or BA, revealed reduced lipid digestion compared to the control product (S/C). In addition, the presence of Si further reduced or delayed lipid digestibility at the end of GID, with a more pronounced effect in products containing EG. This finding could have significant implications for designing functional meat products based on reductions in fat content and lipid absorption, providing groundwork for precision nutrition strategies to improve individual health. Future work in this area should include in vivo validation of lipid digestibility, as well as the assessment of consumer acceptance in larger and more diverse populations.

## 4. Materials and Methods

### 4.1. Materials and Chemicals

For the development of the fat analogs (an emulsion gel, EG, and a fat bulking agent, BA), pork lard (PL), with 99.9% fat content, was obtained after a clarification process of Iberian pork backfat (PB), which was purchased at a local supermarket (Madrid, Spain). Sodium caseinate (SC), with 81% protein content, was provided by Sosa Ingredients S.L. (Barcelona, Spain). The cold gelling system to obtain the gel structures was formed by the hydrocolloid sodium alginate (SA), with 70% carbohydrates, from TRADES (Barcelona, Spain), and tetra-sodium pyrophosphate anhydrous and calcium sulfate from Panreac Química, S.A. (Madrid, Spain). A texturizing mixture (Fibrext 01), containing a blend of fiber, starch, and pea protein, was provided by Sancan (Barcelona, Spain). In addition, the ingredients used to prepare the fresh sausages included pork meat and PB, which were both purchased at a local supermarket (Madrid, Spain). A food-grade preparation of diatomaceous earth powder (DP) “Tierra de Diatomeas^®^”, with a SiO_2_ content of 85%, thus equivalent to 40% Si, was kindly offered by Vitality Gesf S.L. (Valencia, Spain). A commercial seasoning mix (Salch color 1041), containing an appropriate combination of authorized preservatives substances and antioxidants, was provided by Sancan (Barcelona, Spain). Natural lamb casings (Type C-22/24) were supplied by Collelldevall S.L.U. (Girona, Spain). Pepsin (≥2500 U/mg, P7012), pancreatin (8xUSP, P7545), and bile extract (B8631), all of porcine origin, as well as dehydrated calcium chloride, potassium chloride, sodium bicarbonate, and sodium hydrogen carbonate, were all supplied by Sigma Aldrich Chemie GmbH (Steinheim, Germany). Methanol/chloroform were obtained from Fisher Scientific S.L. (Madrid, Spain). Trichloroacetic acid (TCA) was supplied from Panreac Química, SA (Barcelona, Spain), while 2-thiobarbituric acid reagent was acquired from (Merck KGaA, Madrid, Spain). Finally, 1,1,3,3-tetraethoxypropane (TEP) was supplied by Sigma Chemical Co. (St. Louis, MO, USA).

### 4.2. Preparation of Fat Analogs (EG and BA)

Table 7 shows the formulations used to prepare both fat analogs (EG and BA). Briefly, for EG preparation, different solutions were first prepared (12% SC, 5% SA, and other solutions containing 0.15 and 0.10% CaSO_4_ and tetra-sodium pyrophosphate anhydrous, respectively). Then, the SC solution was added to a homogenizer (Thermomix TM 31, Vorwerk España M.S.L., S.C, Madrid, Spain) along with the corresponding amount of Milli-Q water (Table 1) and mixed at ~300 rpm for 30 s. Next, the texturizing mixture was added and mixed at ~1100 rpm for 1 min. To form the primary emulsion, the melted PL was gradually added while increasing the mixing speed from 1100 and 2000 rpm over 3.5 min. The cold gelling system was then added, and the final mixture was further homogenized for 3 min, gradually increasing the speed from 3100 to 4400 rpm. For BA elaboration, the procedure was similar to that of EG, but without the emulsifying protein (SC) solution.

Finally, each type of fat analog was placed in a metal container under pressure to compact it and prevent bubble formation. The samples were then stored in a chilled room at 4 ± 2 °C until their incorporation into the meat products (no more than 4 days). For physicochemical analyses, samples were placed in cylindrical containers (3.5 cm height × 2.5 cm diameter) with lids and stored at 4 ± 2 °C to form the final EG and BA until analysis after 3 days of storage.

### 4.3. Physicochemical Characteristics of PB, PL, and Fat Analogs (EG and BA)

The physicochemical characteristics of the fat analogs, PB, and PL were analyzed after 72 h of chilled storage at 4 °C. Penetration tests were performed using a Texture Analyzer (TA.HDPlus, Stable Micro Systems, Ltd., Godalming, UK), equipped with a 5 kg load cell and Texture Exponent Software (version 6.1.23.0). Samples were penetrated (20 ± 1 °C) as previously described by Cofrades et al. [10]. From the force–distance curves, the following parameters were derived: force at 10 mm (N), total work at 10 mm (mJ), breaking force (N), and breaking work (mJ) (when detected). Color measurements of the lipid materials were conducted on a glass plate using a Konica Minolta CM-3500 D spectrophotometer (Konica Minolta Business Technologies, Tokyo, Japan), set to D65 illuminant/10° observer. Lightness (L*), redness (+a*), and yellowness (+b*) values were recorded to determine color coordinates. All tests were carried out in quintuplicate.

### 4.4. Preparation of Fresh Sausage

Fresh post-rigor meat (30 kg of a mixture of biceps femoris, semimembranosus, semitendinosus, gracilis, and adductor muscles) and PB (6 kg), each from different animals, were obtained from a local market on different days. Both visible fat and connective tissue were removed. Batches of approximately 700 g were vacuum-packed, frozen, and stored at −20 °C until use. Six different batches of fresh sausages were prepared (Table 8) and each formulation was replicated three times. Two batches of control sausages were elaborated exclusively with PB, without and with added DP as a source of Si (S/C and S/C-Si, respectively).

Two batches were prepared in which 75% of PB was replaced with EG, without and with added Si (S/EG and S/EG-Si, respectively). Two more batches were prepared in which 75% of PB was replaced with BA, again without and with Si (S/BA and S/BA-Si, respectively). For the formulation of these sausages, the meat and PB were thawed for 24 h at 4 ± 2 °C. The chilled meat, PB, and EG or BA were minced using a meat mincer equipped with a 6 mm plate (Vam.Dall. Srl., FTSIII, Treviglio, Italy). For all formulations, the required ingredients (Table 2) and 4% commercial seasoning preparation were added to a mixer (MAINCA, Granollers, Barcelona, Spain) and homogenized for a total of 4 min. The mixture was kept at 4 °C for 1.5 h, manually stuffed into 22 mm diameter natural lamb casings, and hand-linked into sausages of 10 ± 2 cm in length. The resulting strings of sausages were weighed, hung, and stored in a chest at 4 ± 2 °C overnight. After overnight storage, each string of sausages was weighed again to calculate processing losses. Individual sausages were then weighed, and vacuum-packaged (Cryovac^®^ OSB3050, Sealead Air, Elmwood Park, NJ, USA) in bags containing 5 sausages each. The packages were stored at 4 ± 2 °C for 12 days. To evaluate the effect of formulation and storage time on quality characteristics, five packages from each treatment were randomly selected for analysis after 0, 5, and 12 days of chilled storage. As this type of sausage is typically consumed cooked, some parameters were analyzed in cooked samples to assess how PB substitution with EG or BA affected quality and lipid in vitro GID. For this purpose, and before analysis, fresh sausages were cooked at 210 °C for 2 min per side using a contact electric grill (Jata classic multigrill model JT950, Navarra, Spain).

### 4.5. Proximate Analysis and Energy Content of Cooked Sausages

Moisture and ash contents of cooked samples were determined according to AOAC methods [38]. Protein was measured with a Nitrogen Determinator LECO FP-2000 (Leco Corporation, St Joseph, MI, USA), while fat content was evaluated following the method by Bligh and Dyer [39]. All tests were performed in triplicate. Energy content was calculated using conversion factors of 4 kcal/g for protein and 9 kcal/g for fat [40].

### 4.6. Physicochemical Parameters of Fresh/Cooked Pork Sausages

#### 4.6.1. Processing, Purge, and Cooking Losses

Processing loss was evaluated by weighing at least five strings of fresh sausages per formulation before and after overnight refrigeration. Results were expressed as a percentage of the initial weight. Purge loss was determined as follows: three bags with 5 sausages (previously weighed) per formulation were tempered for 10 min. The sausages were then removed from the bag, their surfaces were blotted with a paper towel to eliminate surface exudate, and they were weighed. The purge loss was expressed as a percentage of the initial weight. Cooking loss was measured after grilling the sausages to a temperature of 72 °C. At least seven sausages per formulation were weighed before and after cooking to determine the cooking loss by weight difference. Purge and cooking losses were measured after 0, 5, and 12 days of chilled storage.

#### 4.6.2. Texture Profile Analysis (TPA) and Color Measurements of Cooked Pork Sausages

TPA was conducted using the same texture analyzer mentioned above, equipped with a 35 mm diameter cylindrical aluminium probe (P/35). Cylindrical sausage samples with ~20 mm diameter and 20 mm height were compressed twice to 25% of their original height to prevent breakage, with a rest period of 2 s between cycles. The TPA test was performed at room temperature with a trigger force of 0.020 N (2 g) and a test speed of 2 mm/s. The textural properties derived from the force–time curves included hardness (N), springiness (dimensionless), cohesiveness (dimensionless), and chewiness (N). Definitions of TPA parameters can be found in Wee et al. [41]. The color measurements of the cooked sausages were performed on their cross-sectional surfaces at each storage time using the previously mentioned spectrophotometer. Five pieces of sausage per batch were evaluated at 0, 5, and 12 days of chilled storage.

### 4.7. Lipid Oxidation

The thiobarbituric acid-reactive substances (TBARS) method was used to determine (in triplicate) secondary lipid oxidation [10] in both fresh and cooked sausages after 0, 5, and 12 days of chilled storage. Results were expressed as mg MDA/kg sample.

### 4.8. Microbiological Analyses

Microbiological analyses were conducted to determine (in triplicate) total viable count (TVC), lactic acid bacteria (LAB), and *Enterobacteriaceae* levels in fresh sausages during chilled storage, following the methodology described by Pintado et al. [17]. All microbial counts were expressed as logarithms of colony-forming units per gram (Log cfu/g).

### 4.9. Sensory Analysis

Sensory analysis was carried out on day 0. Each grilled sausage was cut into ~4 cm pieces and served immediately after cooking. Sensory evaluation was performed by 33 semi-trained panelists recruited from ICTAN staff. These panelists had prior experience assessing various meat products (hamburgers, frankfurters, pâtés, sausages, etc.) and were widely familiar with both sausages and the attributes tested. A hedonic scale rating test was used to evaluate appearance, acceptability of color, odor, flavor, texture, and overall acceptability for each of the 6 types of sausages. A non-structured scale with fixed extremes (0 = dislike extremely, 9 = like extremely) was used, with each point assigned a corresponding numerical value. All panelists signed a written consent before participation. The study was approved by the CSIC Ethics Committee (063/2019).

### 4.10. In Vitro GID of PB, PL, EG, BA, and Cooked Pork Sausages

EG, BA, and the cooked sausages (S/C, S/C-Si, S/EG, S/EG-Si, S/BA, and S/BA-Si) were subjected to an in vitro INFOGEST 2.0 digestion procedure based on the protocol described by Brodkorb et al. [42], with slight modifications. Each formulation was analyzed within two days of preparation. The complete simulated in vitro GID procedure, including the oral, gastric, and intestinal phases, was performed three times per sample, as described by Cofrades et al. [6,10]. Digest solutions were kept frozen at −20 °C until analysis.

### 4.11. Rate and Extent of Lipolysis of PB, PL, EG, and BA During In Vitro GID

The extent and rate of lipolysis were determined (in triplicate) by measuring the content of free fatty acids (FFA) released during in vitro intestinal digestion. In brief, 0.1 M NaOH was used to neutralize the FFA (pH = 7.0), and the FFA released were monitored using a pH-stat automatic potentiometric titrator (T7, Mettler Toledo, Zurich, Switzerland). Finally, the FFA release rate was calculated using the following equation in accordance with Sarkar et al. [43]:

FFA (%) = (*V*_NaOH_ × *m*_NaOH_ × *M*_lipid_/*W*_lipid_ × 2) × 100 (1)

where *V*_NaOH_ is the volume of NaOH solution required, *m*_NaOH_ is the molarity (0.1 M), *M*_lipid_ is the average molecular weight of lipid, and *W*_lipid_ is the weight of lipid in the mixtures.

### 4.12. Fatty Acid (FA) Profile of the Undigested and Digested Cooked Sausages and Bioaccessibility (BAC) of the Main FA After In Vitro GID

The fatty acid (FA) profile of the undigested cooked sausages was determined (in triplicate) according to the method of Lee et al. [44], as described by Cofrades et al. [10]. FA composition was also evaluated from the micellar fraction obtained after in vitro GID of the different cooked sausages. The final digested solutions of the six types of sausages (in triplicate) were centrifuged (Sorvall Lynx 4000 centrifuge, Thermo Scientific, Waltham, MA, USA) at 12,000 rpm for 30 min at 20 °C. The resulting micellar fraction (the bioaccessible fraction) was collected and used to determine the degree of lipolysis (expressed as the percentage of FFA released), their composition, and the bioaccessibility (BAC) of the main FFA, following the methodology outlined by Cofrades et al. [9,10].

### 4.13. Microstructure Measurements

The microstructure of the digested solutions after 90 min of in vitro GID was determined by confocal laser scanning microscopy (CLSM) using a confocal microscope (Leica TCS SP5 AOBS, Mannheim, Germany) with 20× optics. Fluorophores (Fast Green and Red Nile) were added to a drop (≈10 μL) of sample on a microscope slide, as previously described by Cofrades et al. [6].

### 4.14. Statistical Analysis

A one-way analysis of variance (ANOVA) was performed to evaluate the statistical significance (*p* < 0.05) of the effect of sample formulation. As well, a two-way ANOVA was conducted to evaluate the interaction between formulation and storage time. Both analyses were performed using the SPSS program (v.22, IBM SPSS Inc.; Chicago, IL, USA). Formulation and storage time were considered fixed effects and replication was considered a random effect. The entire experimental design was conducted in triplicate, and differences between replicates were not significant (*p* < 0.05). Results were expressed as mean and standard deviation. Tukey’s HSD test was used to identify significant differences (*p* < 0.05) between formulations and across storage times.

## Figures and Tables

**Table 1:** Physicochemical parameters of pork backfat (PB), pork lard (PL), and fat analogs (EG and BA).

	PB	PL	EG	BA
Texture				
Force at 10 mm (N)	2.83 ± 0.23	0.011 ± 0.00	0.274 ± 0.071 ^B^	0.735 ± 0.09 ^A^
Total work (mJ)	12.0 ± 0.29	0.081 ± 0.01	2.59 ± 0.33 ^B^	5.90 ± 0.43 ^A^
Breaking force (N)	-	-	0.429 ± 0.033 ^B^	1.03 ± 0.06 ^A^
Breaking work (mJ)	-	-	1.05 ± 0.14 ^B^	2.21 ± 0.16 ^A^
Color				
L*	75.05 ± 1.27 ^D^	82.17 ± 0.94 ^C^	90.41 ± 1.51 ^A^	85.25 ± 0.98 ^B^
a*	2.57 ± 0.06 ^A^	−1.92 ± 0.08 ^C^	−1.19 ± 0.68 ^B^	−1.18 ± 0.14 ^B^
b*	3.11 ± 0.07 ^C^	0.992 ± 0.06 ^D^	4.74 ± 0.29 ^A^	4.43 ± 0.28 ^B^

Mean value (*n* = 5) ± standard deviation. ^A–D^ Different letters in the same row indicate significant differences (*p* < 0.05). EG: a fat-in-water emulsion gel prepared with a stabilizer system based on sodium caseinate (SC), a texturizing mixture, and a cold gelling system containing sodium alginate, CaSO_4_, and pyrophosphate. BA: a fat bulking agent stabilized with the same texturizing mixture and cold gelling system used in EG preparation.

**Table 2:** Proximate composition (g/100 g) and energy content (kcal/100 g) of the cooked reduced-fat sausages.

	S/C	S/C-Si	S/EG	S/EG-Si	S/BA	S/BA-Si
Moisture	63.38 ± 0.19 ^A^	62.78 ± 0.17 ^B^	62.37 ± 0.15 ^B^	59.88 ± 0.11 ^C^	63.65 ± 0.46 ^A^	59.97 ± 0.55 ^C^
Protein	18.70 ± 0.56 ^A^	18.13 ± 0.63 ^A^	16.44 ± 0.12 ^B^	16.02 ± 0.28 ^B^	16.14 ± 0.12 ^B^	16.66 ± 0.24 ^B^
Fat	13.11 ± 0.53 ^B^	13.41 ± 0.41 ^B^	15.48 ± 0.51 ^A^	15.30 ± 0.48 ^A^	15.33 ± 0.43 ^A^	15.87 ± 0.28 ^A^
Ash	3.39 ± 0.06 ^B^	5.45 ± 0.11 ^A^	3.42 ± 0.37 ^B^	5.45 ± 0.37 ^A^	3.36 ± 0.02 ^B^	5.71 ± 0.03 ^A^
Energy content	192.79	204.65	205.08	201.78	198.53	209.47

Mean value (n = 3) ± standard deviation. Effect of formulation; S/C: control reduced-fat sausage formulated only with pork backfat (PB); S/C-Si: control reduced-fat sausage formulated only with PB containing diatomaceous earth powder (DP) as a source of silicon (Si); S/EG: reduced-fat sausage formulated by replacing 75% of PB with EG; S/EG-Si: reduced-fat sausage formulated as S/EG containing DP as a source of Si; S/BA: reduced-fat sausage formulated by replacing 75% of PB with BA; S/BA-Si: reduced-fat sausage formulated as S/BA containing DP as a source of Si. ^A–C^ Different letters in the same row indicate significant differences (*p* < 0.05).

**Table 3:** Sensory evaluation of the reduced-fat pork sausages.

Sample	Appearance	Color	Odor	Flavor	Texture	Overall Acceptability
SC	7.77 ± 1.3 ^A^	7.85 ± 1.3 ^A^	7.27 ± 1.5 ^A^	6.67 ± 2.2 ^A,B^	5.83 ± 2.3 ^A^	6.34 ± 2.1 ^A,B^
SC-Si	7.61 ± 1.3 ^A^	7.61 ± 1.4 ^A^	7.91 ± 1.4 ^A^	7.57 ± 1.7 ^A^	7.57 ± 1.7 ^A^	7.16 ± 2.0 ^A^
S/EG	6.65 ± 1.8 ^A^	6.60 ± 2.0 ^A^	7.14 ± 1.8 ^A^	6.87 ± 2.2 ^A,B^	6.88 ± 2.1 ^A^	6.88 ± 2.1 ^A,B^
S/EG-Si	7.21 ± 1.9 ^A^	7.27 ± 1.9 ^A^	7.43 ± 1.6 ^A^	6.64 ± 2.4 ^A,B^	6.02 ± 2.6 ^A^	6.46 ± 2.3 ^A,B^
S/BA	6.47 ± 2.2 ^A^	6.60 ± 2.1 ^A^	6.86 ± 1.9 ^A^	5.75 ± 2.5 ^B^	5.69 ± 2.5 ^A^	5.57 ± 2.5 ^B^
S/BA-Si	7.18 ± 2.3 ^A^	7.25 ± 1.9 ^A^	7.05 ± 2.1 ^A^	6.13 ± 2.3 ^A,B^	6.38 ± 2.2 ^A^	6.26 ± 2.0 ^A,B^

For sample denomination see Table 2. Mean value (n = 33) ± standard deviation. ^A,B^ Different letters in the same column indicate significant differences (*p* < 0.05).

**Table 4:** Changes in the microbial counts (Log cfu/g) of the formulated fresh pork sausages during chilled storage.

	Sample	Storage Time (Days)		
		0	5	12
Total viable count	S/C	5.95 ± 0.01 ^Eb^	6.29 ± 0.03 ^Ca^	6.27 ± 0.04 ^Da^
	S/C-Si	6.31 ± 0.03 ^Db^	6.62 ± 0.15 ^Ba^	6.48 ± 0.04 ^Ba,b^
	S/EG	7.71 ± 0.03 ^Aa,b^	7.84 ± 0.003 ^Aa^	7.58 ± 0.11 ^Ab^
	S/EG-Si	7.56 ± 0.07 ^Bc^	7.93 ± 0.03 ^Aa^	7.71 ± 0.03 ^Ab^
	S/BA	6.66 ± 0.57 ^Ca^	6.67 ± 0.03 ^Ba^	6.44 ± 0.01 ^B,Cb^
	S/BA-Si	6.58 ± 0.02 ^Ca^	6.56 ± 0.01 ^Ba^	6.32 ± 0.04 ^C,Db^
Lactic acid bacteria	S/C	3.95 ± 0.0 ^Cc^	4.26 ± 0.01 ^B,Ca^	4.11 ± 0.07 ^Cb^
	S/C-Si	3.86 ± 0.0 ^Dc^	4.85 ± 0.01 ^Aa^	4.70 ± 0.01 ^Ab^
	S/EG	4.27 ± 0.01 ^Ba^	4.15 ± 0.21 ^Ca^	4.16 ± 0.05 ^Ca^
	S/EG-Si	4.28 ± 0.02 ^Ba^	4.28 ± 0.08 ^B,Ca^	4.19 ± 0.07 ^Ca^
	S/BA	4.34 ± 0.02 ^A,Bb^	4.42 ± 0.01 ^Bb^	4.59 ± 0.10 ^A,Ba^
	S/BA-Si	4.39 ± 0.04 ^Aa^	4.42 ± 0.04 ^Ba^	4.46 ± 0.01 ^Ba^
Enterobacteriaceae	S/C	2.75 ± 0.03 ^Db^	3.06 ± 0.02 ^Ca^	2.76 ± 0.06 ^Cb^
	S/C-Si	2.63 ± 0.01 ^Da,b^	2.76 ± 0.03 ^Ca^	2.50 ± 0.15 ^Db^
	S/EG	4.46 ± 0.08 ^Aa^	4.11 ± 0. 18 ^Ab^	4.04 ± 0.01 ^Ab^
	S/EG-Si	3.99 ± 0.09 ^Ba^	3.59 ± 0.19 ^Bb^	3.25 ± 0.02 ^Bc^
	S/BA	3.98 ± 0.14 ^Aa^	3.97 ± 0.18 ^A,Ba^	3.83 ± 0.10 ^Aa^
	S/BA-Si	3.57 ± 0.22 ^Ca^	3.76 ± 0.10 ^A,Ba^	3.85 ± 0.05 ^Aa^

Mean value (n = 3) ± standard deviation. Effect of formulation; ^A–E^ Different letters in the same column and for the same storage time indicate significant differences (*p* < 0.05). Effect of chilled storage; a–c Different letters in the same row and for the same formulation indicate significant differences (*p* < 0.05).

**Table 5:** Total free fatty acids (FFA) released after in vitro gastrointestinal digestion (GID) and fatty acid (FA) content (mg FA/g fat) of the cooked reduced-fat pork sausages before and after in vitro GID.

		S/C	S/C-Si	S/EG	S/EG-Si	S/BA	S/BA-Si
Total FFA	-	554.02 ± 2.09 ^A^	518.47 ± 5.72 ^B^	512.80 ± 4.20 ^B^	454.82 ± 3.56 ^D^	518.31 ± 1.53 ^B^	500.71 ± 5.06 ^C^
FA profile	Before in vitro GID *	After in vitro GID					
SFA							
Palmitic C16:0	178.83 ± 7.45	71.15 ± 0.98 ^B^	82.58 ± 0.78 ^A^	64.48 ± 0.48 ^D^	53.63 ± 0.61 ^F^	67.07 ± 0.20 ^C^	60.29 ± 1.52 ^E^
Stearic C18:0	98.00 ± 4.58	82.89 ± 0.19 ^A^	76.83 ± 0.86 ^C^	71.93 ± 0.50 ^E^	65.54 ± 0.46 ^F^	79.23 ± 0.25 ^B^	73.04 ± 0.79 ^D^
Other SFA	31.92 ± 1.69	7.74 ± 0.06	8.65 ± 0.09	6.74 ± 0.02	5.81 ± 0.03	6.90 ± 0.21	7.27 ± 0.01
∑SFA	308.75 ± 13.72	161.78 ± 1.24	168.05 ± 1.73	143.15 ± 0.96	124.98 ± 1.10	140.23 ± 2.52	153.57 ± 0.44
MUFA							
Vaccenic C18:1n-7	26.11 ± 1.13	21.61 ± 0.06 ^A^	19.19 ± 0.26 ^D^	21.21 ± 0.20 ^B^	18.31 ± 0.10 ^E^	19.98 ± 0.09 ^C^	20.08 ± 0.11 ^C^
Oleic C18:1n-9	338.8 ± 18.12	281.2 ± 0.74 ^A^	242.0 ± 2.80 ^E^	266.5 ± 2.26 ^C^	237.6 ± 1.81 ^F^	270.6 ± 0.88 ^B^	259.0 ± 1.81 ^D^
Other MUFA	2.36 ± 0.20	23.33 ± 0.04	20.52 ± 0.23	21.00 ± 0.22	17.18 ± 0.13	21.18 ± 0.18	21.38 ± 0.11
∑MUFA	367.27 ± 19.45	326.09 ± 0.83	281.67 ± 3.29	308.75 ± 2.68	273.12 ± 2.04	300.29 ± 2.10	311.96 ± 1.09
PUFA							
Linoleic C18:2n-6	62.40 ± 2.18	53.60 ± 0.09 ^B^	54.65 ± 0.59 ^A^	49.89 ± 0.48 ^C^	46.86 ± 0.38 ^E^	43.40 ± 0.06 ^F^	48.67 ± 0.34 ^D^
Other PUFA	24.30 ± 0.85	12.55 ± 0.08	14.11 ± 0.11	11.02 ± 0.09	9.86 ± 0.05	11.52 ± 0.13	9.39 ± 0.06
∑PUFA	86.70 ± 3.03	66.15 ± 0.01	68.75 ± 0.70	60.91 ± 0.57	56.73 ± 0.43	60.19 ± 0.46	52.79 ± 0.00

* Since the fatty acid profile and fat content are similar in the six sausages, mean values are shown. Mean value (n = 3) ± standard deviation. Effect of formulation; ^A–F^ Different letters in the same row indicate significant differences (*p* < 0.05).

**Table 6:** Bioaccessibility (BAC, %) of the main fatty acids (FA) at the end of in vitro gastrointestinal digestion (GID) for the different cooked pork sausages.

After In Vitro GID	S/C	S/C-Si	S/EG	S/EG-Si	S/BA	S/BA-Si
Main FA						
SFA						
Palmitic C16:0	39.79 ± 0.39 ^B^	46.18 ± 0.31 ^A^	36.05 ± 0.19 ^D^	29.99 ± 0.24 ^F^	37.51 ± 0.08 ^C^	33.71 ± 0.60 ^E^
Stearic C18:0	84.58 ± 0.14 ^A^	78.39 ± 0.62 ^C^	73.40 ± 0.36 ^E^	66.88 ± 0.33 ^F^	80.84 ± 0.18 ^B^	74.53 ± 0.57 ^D^
MUFA						
Vaccenic C18:1n-7	82.74 ± 0.15 ^A^	73.47 ± 0.69 ^D^	81.21 ± 0.54 ^B^	70.11 ± 0.26 ^E^	76.50 ± 0.25 ^C^	76.88 ± 0.30 ^C^
Oleic C18:1n-9	82.99 ± 0.15 ^A^	71.43 ± 0.58 ^E^	78.66 ± 0.47 ^C^	70.13 ± 0.38 ^F^	79.87 ± 0.18 ^B^	76.45 ± 0.38 ^D^
PUFA						
Linoleic C18:2n-6	85.90 ± 0.10 ^B^	87.58 ± 0.67 ^A^	79.95 ± 0.55 ^C^	75.10 ± 0.43 ^E^	69.55 ± 0.07 ^F^	77.99 ± 0.38 ^D^

Mean value (n =3) ± standard deviation. For sample denomination, see Table 2. SFA, saturated fatty acids; MUFA, monounsaturated fatty acids; PUFA, polyunsaturated fatty acid. Effect of formulation; ^A–F^ Different letters in the same row indicate significant differences (*p* < 0.05).

**Table 7:** Ingredients (g/100 g) used in the preparation of the emulsion gel (EG) and the fat bulking agent (BA).

Fat Analog	Sodium Caseinate	Texturizing Mixture	Sodium Alginate	CaSO_4_	Pyrophosphate	Water	Pork lard (PL)
EG	3	2	1	0.15	0.1	53.75	40
BA	0	2	1	0.15	0.1	56.75	40

For sample denomination, see Table 1.

**Table 8:** Ingredients (g/100 g) used in the preparation of fresh pork sausages.

Fresh Sausages	Meat	Pork Backfat (PB)	EG	BA	DP	Water	Seasoning
S/C	60.00	13.00	0	0	0	23.00	4.00
S/C-Si	60.00	13.00	0	0	1.50	21.50	4.00
S/EG	60.00	3.25	21.45	0	0	11.30	4.00
S/EG-Si	60.00	3.25	21.45	0	1.50	9.80	4.00
S/BA	60.00	3.25	0	21.45	0	11.30	4.00
S/BA-Si	60.00	3.25	0	21.45	1.50	9.80	4.00

For sample denomination, see Table 1 and Table 2; DP: diatomaceous earth powder.

## Data Availability

The original contributions presented in this study are included in the article/Supplementary Material. Further inquiries can be directed to the corresponding author.

## Supplementary Materials

The following supporting information can be downloaded at: https://www.mdpi.com/article/10.3390/gels11080618/s1, Figure S1: Force–distance curves of fat analogs (EG and BA) compared to pork backfat (PB) and pork lard (PL).
