# Supplementary material for: Novel Fat Replacers Based on Pork Lard and a Cold Gelling System in the Reformulation of Reduced-Fat Fresh Pork Sausages Containing Silicon from Diatomaceous Earth Powder
Source: Gels. 2025 Aug 8;11(8):618. doi: 10.3390/gels11080618 (PMC12385224; doi:10.3390/gels11080618)
Supplement: Supplementary file 1 [file gels-11-00618-s001.zip › gels-3791951-supplementary.pdf]

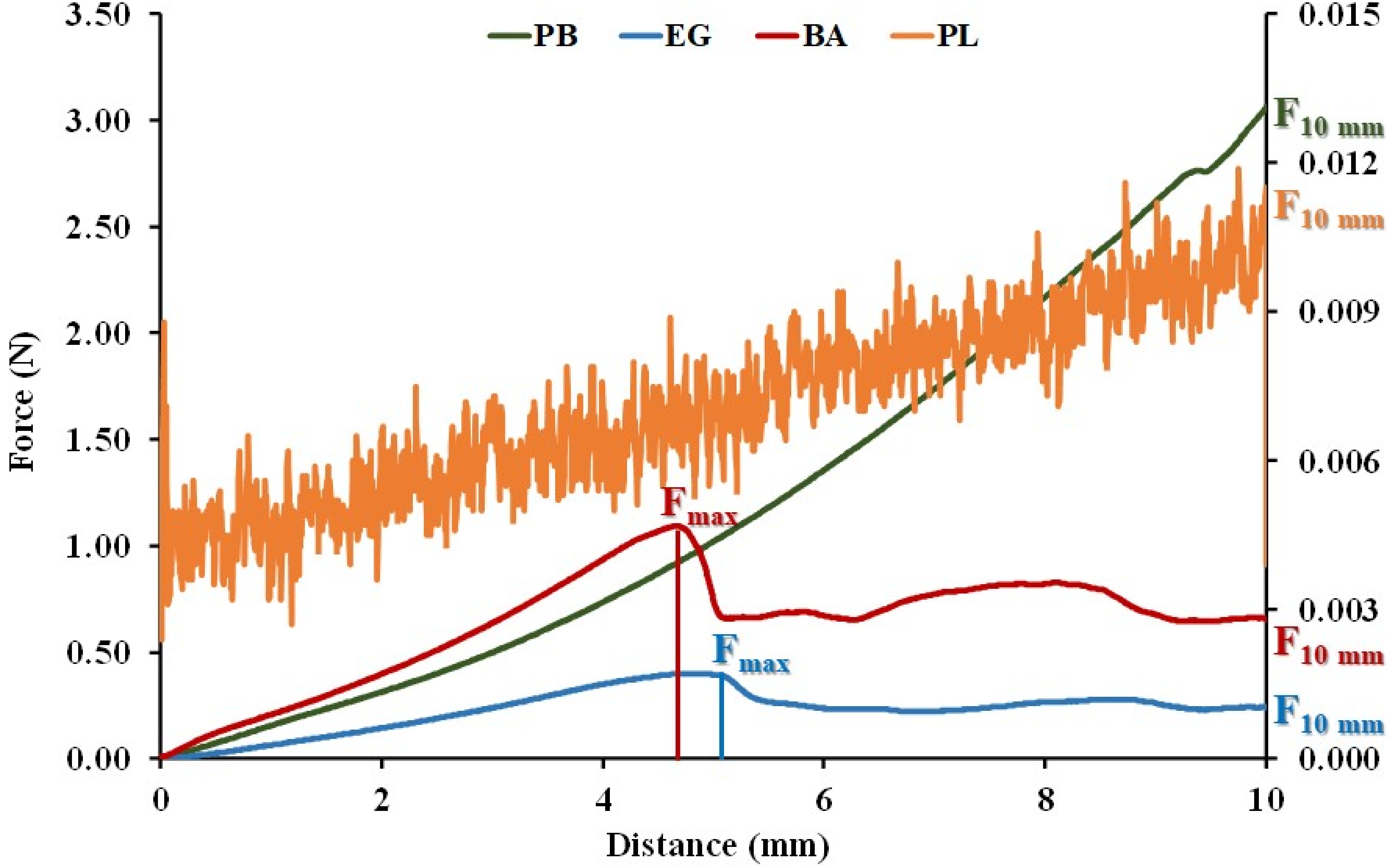

Figure S1. Force-distance curves of fat analogues (EG and BA) as compared to pork backfat (PB) and pork lard (PL).
